# Past, Present, and Future Therapeutic Strategies for NF-1-Associated Tumors

**DOI:** 10.1007/s11912-024-01527-4

**Published:** 2024-05-06

**Authors:** Brian Na, Shilp R. Shah, Harish N. Vasudevan

**Affiliations:** 1 Department of Neurology, UCLA Neuro-Oncology Program, University of California Los Angeles, Los Angeles, CA 90095 USA; 2 Samueli School of Engineering, University of California Los Angeles, Los Angeles, CA 90095 USA; 3 Department of Radiation Oncology, University of California San Francisco, San Francisco, CA 94143 USA; 4 Department of Neurological Surgery, University of California San Francisco, San Francisco, CA 94143 USA

**Keywords:** Neurofibromatosis type 1, Targeted therapy, Cell signaling, MEK inhibition

## Abstract

**Purpose of Review:**

Neurofibromatosis type 1 (NF-1) is a cancer predisposition syndrome caused by mutations in the *NF1* tumor suppressor gene that encodes the neurofibromin protein, which functions as a negative regulator of Ras signaling. We review the past, current, and future state of therapeutic strategies for tumors associated with NF-1.

**Recent Findings:**

Therapeutic efforts for NF-1-associated tumors have centered around inhibiting Ras output, leading to the clinical success of downstream MEK inhibition for plexiform neurofibromas and low-grade gliomas. However, MEK inhibition and similar molecular monotherapy approaches that block Ras signaling do not work for all patients and show limited efficacy for more aggressive cancers such as malignant peripheral nerve sheath tumors and high-grade gliomas, motivating novel treatment approaches.

**Summary:**

We highlight the current therapeutic landscape for NF-1-associated tumors, broadly categorizing treatment into past strategies for serial Ras pathway blockade, current approaches targeting parallel oncogenic and tumor suppressor pathways, and future avenues of investigation leveraging biologic and technical innovations in immunotherapy, pharmacology, and gene delivery.

## Introduction 

Neurofibromatosis type 1 (NF-1) is an autosomal dominant genetic disorder affecting 1 in 3000 individuals caused by germline mutation of the *NF1* gene. The *NF1* gene product neurofibromin is a Ras GTPase-activating protein (RAS-GAP) that converts active GTP-bound Ras into inactive GDP-bound Ras [1–4]. Thus, *NF1* loss leads to constitutive Ras activation and many clinical manifestations of NF-1 such as café-au-lait macules, seizures, chronic pain, vascular issues, bone defects, central and peripheral nervous system tumors, breast cancer, and other malignancies [5, 6]. Of note, tumorigenesis typically requires a second somatic hit and consequent loss of function in the remaining wildtype *NF1* allele [7].

Patients with NF-1 are at significantly increased risk for plexiform neurofibromas (PNs), a benign peripheral nervous system tumor that can transform into malignant peripheral nerve sheath tumors (MPNSTs), and low-grade gliomas (LGGs), a benign central nervous system tumor that can transform into malignant high-grade gliomas (HGGs) [8–10]. In addition, atypical neurofibromatous neoplasms of uncertain biologic potential (ANNUBP) comprise an intermediate tumor entity that reflect the transition from plexiform neurofibromas to MPNSTs [11]. ANNUBPs are associated with *CDKN2AB* loss, and their diagnosis and classification remain an area of active investigation [12–14].

Here, we summarize past, present, and future treatment approaches for NF1-associated tumors. Given neurofibromin’s function as a Ras-GAP and resulting Ras pathway misactivation, therapies to date have primarily focused on inhibiting Ras signaling output at the level of RAF, MEK, ERK, and mTOR [15]. Leveraging our improved understanding of additional genetic hits required for NF1-associated tumorigenesis, more recent work leverages novel pharmacologic approaches to block parallel pathways such as PRC2 or *CDKN2A/B* loss. We conclude with an eye toward the future of NF1 therapeutics currently in preclinical development and early clinical trials including oncolytic viruses, cellular therapy, immune checkpoint inhibitors, gene therapy, and direct Ras inhibition.

## Past Approaches: Serial Inhibition Along the Ras Signaling Axis

**Fig. 1:**
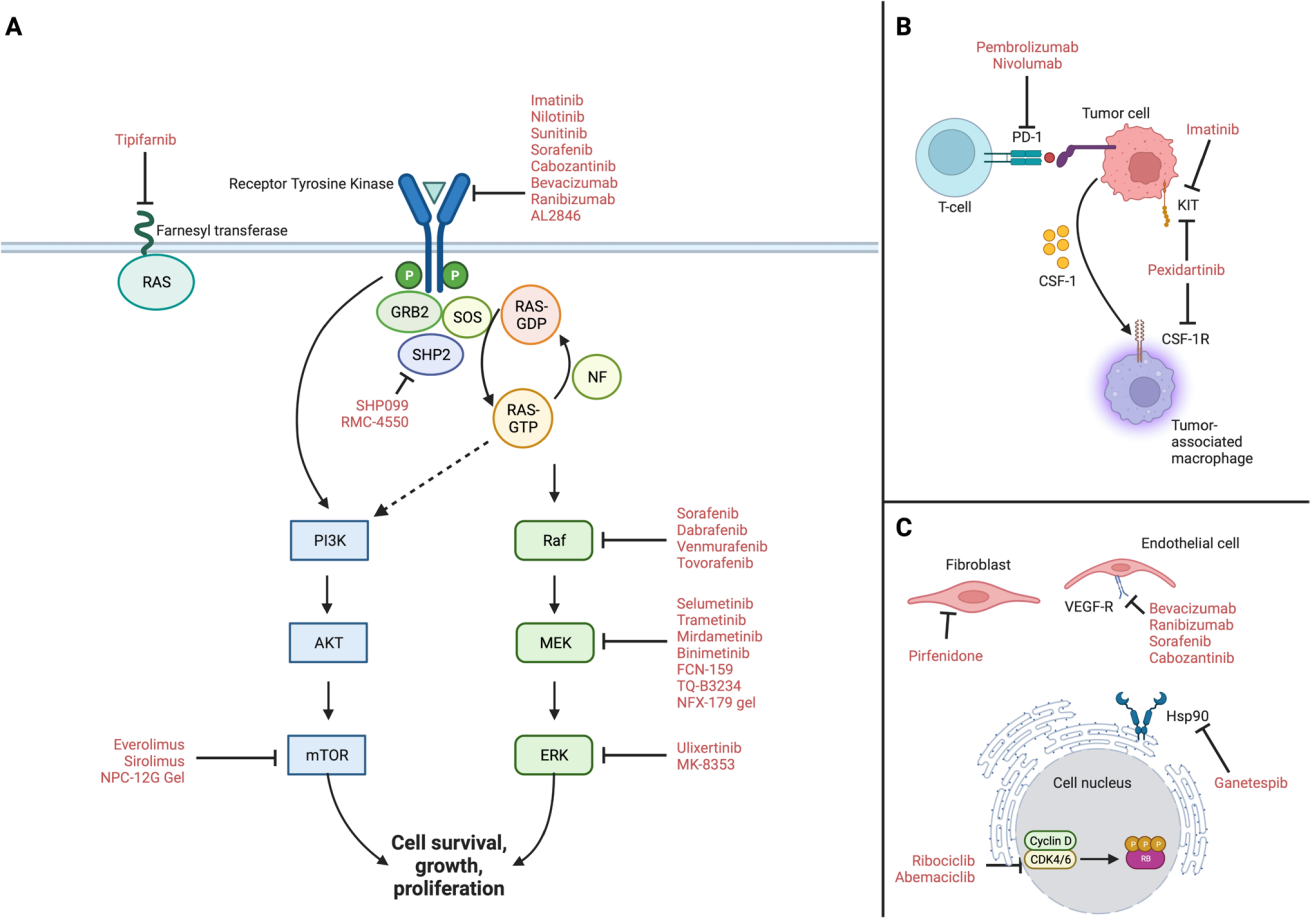
Pathways involved in NF1-associated tumorigenesis. **A** RAF/MEK/ERK inhibitors act on the MAPK pathway; mTOR inhibitors act on the PI3K/AKT/mTOR pathway; receptor tyrosine kinase (RTK) inhibitors and SHP2 inhibitors act on both pathways. Farnesyl transferase inhibitors inhibit RAS signaling. **B** Immunotherapeutic approaches and strategies targeting the tumor microenvironment have been explored, utilizing immune checkpoint, CSF1-R, and KIT inhibitors. **C** Other approaches include targeting other cells within the tumor microenvironment, including fibroblasts and endothelial cells. Cell cycle inhibition utilizing CDK4/6 inhibitors has also been tested

Ras signaling begins at the cell membrane with receptor tyrosine kinase (RTK) activation, setting off a signaling cascade to activate Ras through G-protein exchange factors (GEFs) such as SOS, a process that requires SHP2 and adapter proteins such as GRB2 to promote the formation of active GTP bound Ras (Fig. 1A) [16]. GTP-bound Ras subsequently activates RAF-MEK-ERK while mTOR is classically activated by PI3K signaling, classically through RTK activation with a potential contribution directly by active GTP-bound Ras. Accordingly, upstream RTKs and downstream RAF-MEK-ERK and mTOR have been the primary area of therapeutic investigation to date.

### Mitogen-Activated Protein Kinase Kinase (MEK) Inhibitors (MEKi)

MEK inhibitors (MEKi) have shown significant efficacy for NF1-associated PNs. In particular, the MEKi selumetinib has received FDA approval for symptomatic and inoperable PNs in patients aged 2–18, with ongoing Phase 2 trials in adults displaying similar positive responses [17–19]. The success has spurred the investigation of other MEKi such as binimetinib, cobimetinib, and mirdametinib to enhance clinical efficacy and impact on the tumor microenvironment [20].

Beyond PNs, MEKi is under investigation for additional NF1 manifestations, including atypical neurofibromas, MPNSTs, cutaneous neurofibromas, LGGs, and juvenile myelomonocytic leukemia. In particular, MEKi for cutaneous neurofibromas and LGGs is currently being tested in Phase 2 and 3 trials (NCT03871257, NCT03363217, NCT02285439, NCT03326388, NCT04201457) [21]. Beyond tumor-associated manifestations, MEKi may also have utility for non-tumor manifestations such as pain, bone issues, and neurocognition [17, 18, 22, 23]. Clinical trials indicate reduced pain in PNs, hinting at the MEK pathway’s role in NF1-related pain and suggesting that the tumor microenvironment plays an instrumental function in NF1-PN pathogenesis [17, 18, 23–25]. Despite MEKi’s promise, many challenges remain including dosing strategies, heterogenous responses, treatment resistance, and long-term safety persist, underscoring the need for additional research.

### RAF Inhibition

RAF inhibition, the most proximal downstream signaling protein from RAS, has been studied extensively in *NF1*-mutant tumors. First-generation RAF inhibitors selectively targeting the BRAFV600E mutation show minimal efficacy, with resistance occurring within 6 to 7 months [26, 27]. Accordingly, pan-RAF inhibitors have been developed to address these challenges. Tovorafenib in particular has been investigated in a Phase 2 trial FIREFLY-1 (NCT04775485) for recurrent BRAF-altered pediatric LGGs and demonstrated a meaningful radiographic response, although it is important to note this was not in *NF1* mutant tumors, and further work is necessary to determine whether RAF inhibition alone or in combination with other agents may be useful in the NF-1 setting (NCT04775485) [28•].

### ERK Inhibition

The ERK inhibitor ulixertinib, a novel first-in-class drug exhibiting highly selective, reversible ATP-competitive inhibition of ERK1/2, has demonstrated an antitumor profile for MAPK-activated LGGs [29], and multiple clinical trials testing ulixertinib in the context of *NF1*-deficient cancers are currently underway (NCT05804227, NCT03454035). In addition, preclinical work in mice suggests ERK inhibition may be effective as combination therapy for plexiform neurofibromas [30]. MK-8353 is another ERK1/2 inhibitor that targets both the active and inactive form of ERK [31], but an open-label phase 1b clinical trial investigating the combination therapy of MK-8353 with MEKi selumetinib for advanced solid tumors found unacceptable levels of toxicity at dose levels required for clinical response (NCT03745989) [32]. Additionally, concern has been raised over the long-term effects of both MEK and ERK inhibition on abnormal skeletal manifestations inherent to NF-1 patients. In that regard, the tyrosine kinase inhibitor ponatinib with activity against MEKK2 rescues skeletal defects in vivo, perhaps offering an additional combinatorial strategy to optimize the therapeutic window of ERK inhibitors [33].

### Tyrosine Kinase Inhibitors (TKIs) and SHP2 Inhibition

TKIs disrupt upstream RTK input into Ras signaling, and initial studies with the multi-TKI sunitinib showed reduced tumor burden in a mouse model of NF1-related PNs [34, 35]. However, a subsequent clinical trial was terminated following an adverse event [36]. Furthermore, trials for TKIs imatinib and sorafenib exhibited only modest efficacy in PNs [37, 38]. A more recent Phase 2 trial with the TKI cabozantinib showed promise, with 42% of participants achieving a partial response in progressive PNs [39]. It remains unclear if this effect is mediated directly through RTKs or via alternate pathways, as preclinical work indicates that cabozantinib activity against MAPK interacting kinases (MNKs), when combined with the MEKi mirdametinib, induces regression in a genetically engineered mouse malignant peripheral nerve sheath tumor (MPNST) model [40].

Another approach to modulate upstream inputs is through SHP2, which potentiates Ras GTP loading, and thus, SHP2 inhibitors (SHP2i) may offer a promising approach for NF-1-associated tumors [41]. Indeed, *NF1*-mutant neuroblastomas are sensitive to the SHP2 inhibitor SHP099, and the combination of MEKi/SHP2i demonstrated improved efficacy across multiple preclinical models [42, 43]. By targeting signaling proteins upstream within the RAS-MAPK pathway, SHP2 inhibition may potentiate other targeted therapies in NF-1-associated tumors.

### Mammalian Target of Rapamycin (mTOR) Inhibitors

The mTOR pathway is hyperactivated in *NF1*-deficient tumors [8, 44, 45]. Sirolimus, an FDA-approved mTOR inhibitor, was tested for PNs in a Phase 2 clinical trial, leading to increased time to progression but no significant difference in tumor volume [46, 47]. Similarly, everolimus was studied in a Phase 2 trial and showed no efficacy for NF1-related PNs but exhibited a significant radiographic reduction in recurrent NF1-LGGs, perhaps underscoring heterogeneity between different NF1 tumor entities [48, 49]. A recently completed Phase 2 trial investigated a combination therapy of sirolimus plus MEKi for unresectable or metastatic MPNSTs, and final trial results are eagerly anticipated [50].

## Present Approaches: Parallel Inhibition of Co-mutated Tumor Suppressors

Following *NF1* loss, additional genetic hits are required for malignant transformation of benign nervous system tumors into their malignant entities [51, 52]. Of these, *CDKN2A/B* loss and PRC2 loss are well-appreciated steps in the transition from PN to MPNST [53, 54]. Loss of the tumor suppressor *CDKN2A/B*, which is associated with transition from PN to ANNUBP, leads to cyclin-dependent kinase (CDK) activation, motivating the application of CDK4/6 inhibitors (CDK4/6i) in NF1-associated tumors [55]. With respect to PRC2, *SUZ12* and *EED*, obligate members of the PRC2 epigenetic complex, are recurrently mutated in MPNSTs but not PNs [54].

### CDK4/6 Inhibition (CDK4/6i)

The CDK4/6i abemaciclib demonstrated synergistic anti-tumor effects when combined with the ERKi LY3214996 for PN treatment in vivo [30]. A clinical trial (NCT04000529) is ongoing to evaluate the safety and efficacy of ribociclib combined with the SHP2 inhibitor TNO155 for advanced solid tumors. In *NF1*-mutant breast cancer, the CDK4/6i palbociclib reduced growth and enhanced sensitivity to the antiestrogenic medication fulvestrant, indicating a synergistic relationship [56, 57]. These findings suggest that CDK4/6 inhibition combined with targeted therapies may offer an improved treatment strategy.

### Targeting Polycomb Repressive Complex 2 (PRC2)

PRC2 loss through mutation of its obligate members *SUZ12* or *EED* is common and provides a rationale for targeted combination therapies of NF1-associated tumors with bromodomain inhibitors [54, 58, 59]. The bromodomain protein BRD4 plays a crucial role in NF1-associated MPNST development and comprises a therapeutic target to potentially overcome MEKi resistance [60]. Interestingly, MPNSTs depleted of BRD4 protein exhibit a strong cytotoxic response to the pan-BET bromodomain inhibitor JQ1 [61]. Additionally, suppressing *SUZ12* enhances the impact of PD-901/JQ1 administration in NF1-deficient cells [62]. In a study on *NF1*-mutated ovarian cancer, co-administration of JQ1 and MEKi trametinib proved effective in overcoming the common rapid drug resistance associated with single-agent MEKi [63]. A second bromodomain inhibitor, bromosporine, demonstrated a superior therapeutic index when combined with MEKi cobimetinib for treating immunotherapy-resistant *NF1*-mutant melanoma, compared to MEKi treatment alone [64•]. More recent work suggests DNMT1 inhibition may be a druggable dependency upon PRC2 loss, providing yet another targeted approach [65]. Overall, targeting PRC2 loss holds significant promise for enhancing existing strategies for *NF1*-deficient tumors by increasing cytotoxicity and limiting the development of drug resistance.

## Future Therapeutic Approaches: Beyond Targeted Therapeutics

Decades of work understanding the genetic and signaling mechanisms underlying *NF1-*associated tumorigenesis have nominated numerous targets, yet there remains an urgent, unmet clinical need for new therapies with improved therapeutic windows and more durable responses. Promising preclinical approaches leveraging pharmacologic advances to investigate gene therapy, directly targeting Ras, or reestablishing immune system function with cellular CAR-T therapies, checkpoint inhibitors, or oncolytic viral therapy (Fig. 1B) are areas of active investigation that offer potential for the next generation of therapeutics.

### Gene Therapy

Gene therapy through adeno-associated viral (AAV) vectors offers a potentially curative approach aimed at *NF1* gene reconstitution. Although full-length reconstitution has been historically limited by the size of the *NF1* gene, the neurofibromin GTPase-activating protein-related domain (GRD) alone, fused with an H-Ras C10 sequence, demonstrates potent ERK1/2 suppression, reduced cell growth, and exhibiting specificity for *NF1-*mutant MPNST cells compared to *NF1-*intact cells [66, 67]. However, numerous open questions remain regarding gene targeting specificity, efficient delivery, and maximum therapeutic payload size that require further research to harness the potential of neurofibromin reconstitution.

### Direct Ras Inhibition

Although Ras was historically considered to be undruggable as a direct pharmacologic target, multiple covalent inhibitors targeting oncogenic Ras variants now exist. In NF-1-associated tumors lacking an oncogenic Ras variant, multiple levels of evidence support a critical role for KRAS in mediating the effects of *NF1* loss [68, 69]. Accordingly, recently described pan KRAS inhibitors that inhibit wildtype KRAS yet spare NRAS and HRAS may show therapeutic efficacy for *NF1* mutant tumors [70•]. However, whether KRAS is the critical Ras effector for all NF-1 manifestations remains unclear. Moreover, blocking Ras alone may be insufficient, and thus, combination approaches with existing therapies may be required to overcome resistance. Indeed, treatment resistance is a recognized problem for KRAS G12C inhibitors [71]. SHP2 inhibition has shown synergy with KRAS^G12C ^inhibitors [72, 73]. SHP2 inhibition prevents the action of SOS1/2, increasing the amount of the GDP-bound state of KRAS^G12C ^which is the target of KRAS inhibitors [74]. This is supported by the findings of KRAS-amplified cancer cell lines exhibiting increased sensitivity to SHP2 inhibition [72, 75].

### CAR-T Cell Therapy

CAR-T cell therapy engineers T-cells with the ability to target overexpressed antigens specific to cancer cells and has revolutionized the treatment of cancer types, primarily hematologic malignancies such as leukemia and lymphoma [76]. Ongoing clinical trials (NCT03618381) are investigating EGFR-targeting CAR-T cell therapy for MPNSTs, and CAR-T therapy for *NF1*-mutated high-grade gliomas using tumor-specific internal peptides is being tested to address the challenge of non-unique expression on the surface of solid tumors. While many questions remain, including the competency of T cells derived from patients harboring a germline *NF1* mutation, [77] CAR-T cell therapy is a promising area of investigation for *NF1*-mutant tumors.

### Immune Checkpoint Inhibitors (ICI)

ICIs have revolutionized cancer care for multiple solid tumor types, and case reports suggest potential ICI efficacy in patients with MPNSTs [78]. The PD-1 inhibitor pembrolizumab was investigated in an MPMS clinical trial but was closed due to limited accrual (NCT02691026). Ongoing clinical trials are evaluating the efficacy of adjuvant nivolumab along with CTLA-4 checkpoint inhibitor ipilimumab for newly diagnosed MPNSTs (NCT04465643, NCT02834013).

Beyond PD-1 axis blockade, colony-stimulating factor-1 receptor (CSF-1R) is often upregulated in various cancer phenotypes and plays a critical role in macrophage polarization, converting tumor-associated macrophages from the tumoricidal M0 or M1 phenotype to the tumorigenic M2 phenotype [79]. Pexidartinib, a novel small molecule CSF-1R inhibitor, showed promising results in a Phase 1 study for MPNSTs when combined with sirolimus, and a Phase 2 trial is now underway (NCT02584647) [80]. MK-1775, another novel ICI, is being investigated for combating MPNSTs by inhibiting *WEE1*, a key regulator of cell cycle progression [81].

### Oncolytic Viral (OV) Therapy

OV therapy is another promising approach for *NF1*-associated tumors. A measles virus-based OV approach shows efficacy in MPNST cells [82], leading to a Phase 1 trial underway to investigate the clinical efficacy of this technique (NCT02700230). Other trials leverage alternate viral agents such as Herpes Simplex Virus (HSV) HSV1716 to preferentially target actively dividing nervous system tumor cells (NCT00931931).

## Conclusion

Patients with NF-1 can exhibit a diverse array of clinical manifestations. Building on classic NF1/Ras biology, MEK inhibitors are an effective therapy for a number of NF1-related manifestations, yet the heterogeneity and durability of their response motivate the development of additional approaches. Ongoing research into biologic mechanisms and signal transduction pathways dysregulated in *NF1*-associated tumors holds the potential to reveal additional therapeutic vulnerabilities. Moreover, targeting the tumor microenvironment and employing combination molecular therapies show promise. Continuous investigation through mechanistic investigation, preclinical modeling, clinical trials, the accumulation of long-term safety data, and collaboration between basic scientists and clinicians will be pivotal in advancing therapeutic interventions for NF1-associated tumors.

## Data Availability

No datasets were generated or analysed during the current study.

## References

[1] Anderson JL, Gutmann DH. Neurofibromatosis type 1. Handb Clin Neurol. 2015;132:75–86. 26564071 10.1016/B978-0-444-62702-5.00004-4

[2] Martin GA, et al. The GAP-related domain of the neurofibromatosis type 1 gene product interacts with ras p21. Cell. 1990;63:843–9. 2121370 10.1016/0092-8674(90)90150-D

[3] Basu TN, et al. Aberrant regulation of ras proteins in malignant tumour cells from type 1 neurofibromatosis patients. Nature. 1992;356:713–5. 1570015 10.1038/356713a0

[4] DeClue JE, et al. Abnormal regulation of mammalian p21ras contributes to malignant tumor growth in von Recklinghausen (type 1) neurofibromatosis. Cell. 1992;69:265–73. 1568246 10.1016/0092-8674(92)90407-4

[5] Trovó-Marqui A, Tajara E. Neurofibromin: a general outlook. Clin Genet. 2006;70:1–13. 16813595 10.1111/j.1399-0004.2006.00639.x

[6] Gutmann DH, et al. Neurofibromatosis type 1. Nat Rev Dis Primer. 2017;3:17004. 10.1038/nrdp.2017.4 28230061

[7] Spyk SL, Thomas N, Cooper DN, Upadhyaya M. Neurofibromatosis type 1-associated tumours: their somatic mutational spectrum and pathogenesis. Hum Genomics. 2011;5:623–90. 22155606 10.1186/1479-7364-5-6-623 PMC3525246

[8] Le LQ, et al. Susceptible stages in Schwann cells for NF1-associated plexiform neurofibroma development. Cancer Res. 2011;71:4686–95. 21551250 10.1158/0008-5472.CAN-10-4577 PMC3145496

[9] Zhu Y, Ghosh P, Charnay P, Burns DK, Parada LF. Neurofibromas in NF1: Schwann cell origin and role of tumor environment. Science. 2002;296:920–2. 11988578 10.1126/science.1068452 PMC3024710

[10] Packer RJ, et al. Implications of new understandings of gliomas in children and adults with NF1: report of a consensus conference. Neuro-Oncol. 2020;22:773–84. 32055852 10.1093/neuonc/noaa036 PMC7283027

[11] Kresbach C, et al. Atypical neurofibromas reveal distinct epigenetic features with proximity to benign peripheral nerve sheath tumor entities. Neuro-Oncol. 2023;25:1644–55. 36866403 10.1093/neuonc/noad053 PMC10479771

[12] • Rhodes SD, et al. Cdkn2a (Arf) loss drives NF1-associated atypical neurofibroma and malignant transformation. Hum Mol Genet. 2019;28:2752–62. (**This is an important study that defines the molecular mechanisms underlying malignant transformation of NF-1-associated plexiform neurofibromas to malignant peripheral nerve sheath tumors.**) 31091306 10.1093/hmg/ddz095 PMC6687955

[13] Tong S, Devine WP, Shieh JT. Tumor and constitutional sequencing for neurofibromatosis type 1. JCO Precis Oncol. 2022;6:e2100540. 35584348 10.1200/PO.21.00540 PMC9200388

[14] Mitchell DK, et al. Spatial gene expression profiling unveils immuno-oncogenic programs of NF1-associated peripheral nerve sheath tumor progression. Clin Cancer Res Off J Am Assoc Cancer Res. 2023. 10.1158/1078-0432.CCR-23-2548. 10.1158/1078-0432.CCR-23-2548 PMC11095977 38127282

[15] Kahen EJ, et al. Neurofibromin level directs RAS pathway signaling and mediates sensitivity to targeted agents in malignant peripheral nerve sheath tumors. Oncotarget. 2018;9:22571–85. 29854299 10.18632/oncotarget.25181 PMC5978249

[16] Simanshu DK, Nissley DV, McCormick F. RAS proteins and their regulators in human disease. Cell. 2017;170:17–33. 28666118 10.1016/j.cell.2017.06.009 PMC5555610

[17] Dombi E, et al. Activity of selumetinib in neurofibromatosis type 1-related plexiform neurofibromas. N Engl J Med. 2016;375:2550–60. 28029918 10.1056/NEJMoa1605943 PMC5508592

[18] • Gross AM, et al. Selumetinib in children with inoperable plexiform neurofibromas. N Engl J Med. 2020;382:1430–42. (**This clinical trial evaluating the efficacy of the MEK inhibitor selumetinib led to the FDA approval of selumetinib for symptomatic, inoperable plexiform neurofibromas in NF-1 pediatric patients.**) 32187457 10.1056/NEJMoa1912735 PMC7305659

[19] O’Sullivan Coyne GH, et al. Phase II trial of the MEK 1/2 inhibitor selumetinib (AZD6244, ARRY-142886 Hydrogen Sulfate) in adults with neurofibromatosis type 1 (NF1) and inoperable plexiform neurofibromas (PN). J Clin Oncol. 2020;38:3612–3612. 10.1200/JCO.2020.38.15_suppl.3612

[20] Bendell JC, et al. A phase 1 dose-escalation and expansion study of binimetinib (MEK162), a potent and selective oral MEK1/2 inhibitor. Br J Cancer. 2017;116:575–83. 28152546 10.1038/bjc.2017.10 PMC5344293

[21] Fangusaro J, et al. Selumetinib in paediatric patients with BRAF-aberrant or neurofibromatosis type 1-associated recurrent, refractory, or progressive low-grade glioma: a multicentre, phase 2 trial. Lancet Oncol. 2019;20:1011–22. 31151904 10.1016/S1470-2045(19)30277-3 PMC6628202

[22] Ma Y, et al. A molecular basis for neurofibroma-associated skeletal manifestations in NF1. Genet Med Off J Am Coll Med Genet. 2020;22:1786–93. 10.1038/s41436-020-0885-3 PMC8106869 32601387

[23] Walsh KS, et al. Impact of MEK inhibitor therapy on neurocognitive functioning in NF1. Neurol Genet. 2021;7:e616. 34377779 10.1212/NXG.0000000000000616 PMC8351286

[24] Ciruela A, et al. Identification of MEK1 as a novel target for the treatment of neuropathic pain. Br J Pharmacol. 2003;138:751–6. 12642375 10.1038/sj.bjp.0705103 PMC1573714

[25] Ji RR, Baba H, Brenner GJ, Woolf CJ. Nociceptive-specific activation of ERK in spinal neurons contributes to pain hypersensitivity. Nat Neurosci. 1999;2:1114–9. 10570489 10.1038/16040

[26] Song Y, et al. Targeting RAS–RAF–MEK–ERK signaling pathway in human cancer: Current status in clinical trials. Genes Dis. 2022;10:76–88. 37013062 10.1016/j.gendis.2022.05.006 PMC10066287

[27] Degirmenci U, Yap J, Sim YRM, Qin S, Hu J. Drug resistance in targeted cancer therapies with RAF inhibitors. Cancer Drug Resist. 2021;4:665–83. 35582307 10.20517/cdr.2021.36 PMC9094075

[28] • Kilburn LB, et al. The type II RAF inhibitor tovorafenib in relapsed/refractory pediatric low-grade glioma: the phase 2 FIREFLY-1 trial. Nat Med. 2024;30:207–17. (**This is an important clinical trial demonstrating response in heavily pre-treated pediatric patients with BRAF-altered low-grade glioma that has implications for patients with NF1-associated low-grade gliomas.**) 37978284 10.1038/s41591-023-02668-y PMC10803270

[29] Sigaud R, et al. The first-in-class ERK inhibitor ulixertinib shows promising activity in mitogen-activated protein kinase (MAPK)-driven pediatric low-grade glioma models. Neuro-Oncol. 2022;25:566–79. 10.1093/neuonc/noac183 PMC10013652 35882450

[30] Flint AC, et al. Combined CDK4/6 and ERK1/2 inhibition enhances antitumor activity in NF1-associated plexiform neurofibroma. Clin Cancer Res. 2023;29:3438–56. 37406085 10.1158/1078-0432.CCR-22-2854 PMC11060649

[31] Boga SB, et al. MK-8353: Discovery of an orally bioavailable dual mechanism ERK inhibitor for oncology. ACS Med Chem Lett. 2018;9:761–7. 30034615 10.1021/acsmedchemlett.8b00220 PMC6047169

[32] Stathis A, et al. Results of an open-label phase 1b study of the ERK inhibitor MK-8353 plus the MEK inhibitor selumetinib in patients with advanced or metastatic solid tumors. Invest New Drugs. 2023;41:1–11. 37040046 10.1007/s10637-022-01326-3 PMC10289957

[33] Bok S, et al. MEKK2 mediates aberrant ERK activation in neurofibromatosis type I. Nat Commun. 2020;11:5704. 33177525 10.1038/s41467-020-19555-6 PMC7658220

[34] Yang Y, Li S, Wang Y, Zhao Y, Li Q. Protein tyrosine kinase inhibitor resistance in malignant tumors: molecular mechanisms and future perspective. Signal Transduct Target Ther. 2022;7:1–36. 36115852 10.1038/s41392-022-01168-8 PMC9482625

[35] Ferguson MJ, et al. Preclinical evidence for the use of sunitinib malate in the treatment of plexiform neurofibromas. Pediatr Blood Cancer. 2016;63:206–13. 26375012 10.1002/pbc.25763 PMC4862309

[36] Study Details | Study of Sutent®/Sunitinib (SU11248) in subjects with NF-1 plexiform neurofibromas | ClinicalTrials.gov. https://clinicaltrials.gov/study/NCT01402817.

[37] Robertson KA, et al. Imatinib mesylate for plexiform neurofibromas in patients with neurofibromatosis type 1: a phase 2 trial. Lancet Oncol. 2012;13:1218–24. 23099009 10.1016/S1470-2045(12)70414-X PMC5380388

[38] Kim A, et al. Phase I trial and pharmacokinetic study of sorafenib in children with neurofibromatosis type I and plexiform neurofibromas. Pediatr Blood Cancer. 2013;60:396–401. 22961690 10.1002/pbc.24281 PMC6309697

[39] Solares I, Viñal D, Morales-Conejo M, Rodriguez-Salas N, Feliu J. Novel molecular targeted therapies for patients with neurofibromatosis type 1 with inoperable plexiform neurofibromas: a comprehensive review. ESMO Open. 2021;6:100223. 34388689 10.1016/j.esmoop.2021.100223 PMC8363824

[40] Lock R, et al. Cotargeting MNK and MEK kinases induces the regression of NF1-mutant cancers. J Clin Invest. 2016;126:2181–90. 27159396 10.1172/JCI85183 PMC4887164

[41] Chen Y-NP, et al. Allosteric inhibition of SHP2 phosphatase inhibits cancers driven by receptor tyrosine kinases. Nature. 2016;535:148–52. 27362227 10.1038/nature18621

[42] Cai J, et al. High-risk neuroblastoma with NF1 loss of function is targetable using SHP2 inhibition. Cell Rep. 2022;40:111095. 35905710 10.1016/j.celrep.2022.111095 PMC10353975

[43] Wang J, et al. Combined inhibition of SHP2 and MEK is effective in models of NF1-deficient malignant peripheral nerve sheath tumors. Cancer Res. 2020;80:5367–79. 33032988 10.1158/0008-5472.CAN-20-1365 PMC7739379

[44] Johannessen CM, et al. The NF1 tumor suppressor critically regulates TSC2 and mTOR. Proc Natl Acad Sci U S A. 2005;102:8573–8. 15937108 10.1073/pnas.0503224102 PMC1142482

[45] Dasgupta B, Yi Y, Chen DY, Weber JD, Gutmann DH. Proteomic analysis reveals hyperactivation of the mammalian target of rapamycin pathway in neurofibromatosis 1-associated human and mouse brain tumors. Cancer Res. 2005;65:2755–60. 15805275 10.1158/0008-5472.CAN-04-4058

[46] Weiss B, et al. Sirolimus for non-progressive NF1-associated plexiform neurofibromas: an NF clinical trials consortium phase II study. Pediatr Blood Cancer. 2014;61:982–6. 24851266 10.1002/pbc.24873

[47] Weiss B, et al. Sirolimus for progressive neurofibromatosis type 1–associated plexiform neurofibromas: a Neurofibromatosis Clinical Trials Consortium phase II study. Neuro-Oncol. 2015;17:596–603. 25314964 10.1093/neuonc/nou235 PMC4483073

[48] Zehou O, et al. Absence of efficacy of everolimus in neurofibromatosis 1-related plexiform neurofibromas: results from a phase 2a trial. J Invest Dermatol. 2019;139:718–20. 30339775 10.1016/j.jid.2018.09.016

[49] Ullrich NJ, et al. A phase II study of continuous oral mTOR inhibitor everolimus for recurrent, radiographic-progressive neurofibromatosis type 1–associated pediatric low-grade glioma: a Neurofibromatosis Clinical Trials Consortium study. Neuro-Oncol. 2020;22:1527–35. 32236425 10.1093/neuonc/noaa071 PMC7566451

[50] Study Details | SARC031: MEK inhibitor selumetinib (AZD6244) in combination with the mTOR inhibitor sirolimus for patients with malignant peripheral nerve sheath tumors | ClinicalTrials.gov. https://clinicaltrials.gov/study/NCT03433183.

[51] Vasudevan HN, et al. Functional interactions between neurofibromatosis tumor suppressors underlie Schwann cell tumor de-differentiation and treatment resistance. Nat Commun. 2024;15:477. 38216572 10.1038/s41467-024-44755-9 PMC10786885

[52] Lobbous M, et al. An update on neurofibromatosis type 1-associated gliomas. Cancers. 2020;12:114. 31906320 10.3390/cancers12010114 PMC7017116

[53] Pemov A, et al. Low mutation burden and frequent loss of CDKN2A/B and SMARCA2, but not PRC2, define premalignant neurofibromatosis type 1–associated atypical neurofibromas. Neuro-Oncol. 2019;21:981–92. 30722027 10.1093/neuonc/noz028 PMC6682216

[54] Lee W, et al. PRC2 is recurrently inactivated through EED or SUZ12 loss in malignant peripheral nerve sheath tumors. Nat Genet. 2014;46:1227–32. 25240281 10.1038/ng.3095 PMC4249650

[55] Williams KB, Largaespada DA. New model systems and the development of targeted therapies for the treatment of neurofibromatosis type 1-associated malignant peripheral nerve sheath tumors. Genes. 2020;11:477. 32353955 10.3390/genes11050477 PMC7290716

[56] Cristofanilli M, et al. Overall survival with palbociclib and fulvestrant in women with HR+/HER2− ABC: updated exploratory analyses of PALOMA-3, a double-blind, phase iii randomized study. Clin Cancer Res. 2022;28:3433–42. 35552673 10.1158/1078-0432.CCR-22-0305 PMC9662922

[57] Pearson A, et al. Inactivating NF1 mutations are enriched in advanced breast cancer and contribute to endocrine therapy resistance. Clin Cancer Res. 2020;26:608–22. 31591187 10.1158/1078-0432.CCR-18-4044

[58] De Raedt T, et al. PRC2 loss amplifies Ras-driven transcription and confers sensitivity to BRD4-based therapies. Nature. 2014;514:247–51. 25119042 10.1038/nature13561

[59] Zhang M, et al. Somatic mutations of SUZ12 in malignant peripheral nerve sheath tumors. Nat Genet. 2014;46:1170–2. 25305755 10.1038/ng.3116 PMC4383254

[60] Patel AJ, et al. BET bromodomain inhibition triggers apoptosis of NF1-associated malignant peripheral nerve sheath tumors through bim induction. Cell Rep. 2014;6:81–92. 24373973 10.1016/j.celrep.2013.12.001 PMC3904298

[61] Cooper JM, et al. Overcoming BET inhibitor resistance in malignant peripheral nerve sheath tumors. Clin Cancer Res Off J Am Assoc Cancer Res. 2019;25:3404–16. 10.1158/1078-0432.CCR-18-2437 PMC6548569 30796033

[62] Zhang X, Murray B, Mo G, Shern JF. The role of polycomb repressive complex in malignant peripheral nerve sheath tumor. Genes. 2020;11:287. 32182803 10.3390/genes11030287 PMC7140867

[63] Kurimchak AM, et al. Intrinsic resistance to MEK inhibition through BET protein mediated kinome reprogramming in NF1-deficient ovarian cancer. Mol Cancer Res MCR. 2019;17:1721–34. 31043489 10.1158/1541-7786.MCR-18-1332 PMC6679760

[64] • Dar AA, et al. Bromodomain inhibition overcomes treatment resistance in distinct molecular subtypes of melanoma. Proc Natl Acad Sci U S A. 2022;119:e2206824119. (**This is an important preclinical study that demonstrates that treatment-naïve and treatment-resistant NF1-mutated melanomas respond to a combination of bromosporine and cobinimetinib, suggesting a combination of MEK and bromodomain inhibition may show efficacy in NF-1-associated tumors.**) 35969744 10.1073/pnas.2206824119 PMC9407673

[65] Patel AJ, et al. PRC2-inactivating mutations in cancer enhance cytotoxic response to DNMT1-targeted therapy via enhanced viral mimicry. Cancer Discov. 2022;12:2120–39. 35789380 10.1158/2159-8290.CD-21-1671 PMC9437570

[66] Bai R-Y, et al. Feasibility of using NF1-GRD and AAV for gene replacement therapy in NF1-associated tumors. Gene Ther. 2019;26:277–86. 31127187 10.1038/s41434-019-0080-9 PMC6588423

[67] Leier A, et al. Mutation-directed therapeutics for neurofibromatosis type I. Mol Ther Nucleic Acids. 2020;20:739–53. 32408052 10.1016/j.omtn.2020.04.012 PMC7225739

[68] Dasgupta B, Li W, Perry A, Gutmann DH. Glioma formation in neurofibromatosis 1 reflects preferential activation of K-RAS in astrocytes. Cancer Res. 2005;65:236–45. 15665300 10.1158/0008-5472.236.65.1

[69] Khalaf WF, et al. K-ras is critical for modulating multiple c-kit-mediated cellular functions in wild-type and Nf1+/- mast cells. J Immunol Baltim Md. 2007;1950(178):2527–34. 10.4049/jimmunol.178.4.2527 17277161

[70] • Kim D, et al. Pan-KRAS inhibitor disables oncogenic signalling and tumour growth. Nature. 2023;619:160–6. (**This study demonstrates that pharmacologic agents inhibiting wild-type KRAS while sparing other Ras proteins can show efficacy in tumor models.**) 37258666 10.1038/s41586-023-06123-3 PMC10322706

[71] Awad MM, et al. Acquired resistance to KRASG12C inhibition in cancer. N Engl J Med. 2021;384:2382–93. 34161704 10.1056/NEJMoa2105281 PMC8864540

[72] Li T, et al. Developing SHP2-based combination therapy for KRAS-amplified cancer. JCI Insight. 2023;8:e152714. 10.1172/jci.insight.152714 PMC9977440 36752207

[73] Hallin J, et al. The KRASG12C inhibitor, MRTX849, provides insight toward therapeutic susceptibility of KRAS mutant cancers in mouse models and patients. Cancer Discov. 2020;10:54–71. 31658955 10.1158/2159-8290.CD-19-1167 PMC6954325

[74] Fedele C, et al. SHP2 inhibition diminishes KRASG12C cycling and promotes tumor microenvironment remodeling. J Exp Med. 2020;218:e20201414. 10.1084/jem.20201414 PMC7549316 33045063

[75] Drilon A, et al. SHP2 inhibition sensitizes diverse oncogene-addicted solid tumors to re-treatment with targeted therapy. Cancer Discov. 2023;13:1789–801. 37269335 10.1158/2159-8290.CD-23-0361 PMC10401072

[76] June CH, O’Connor RS, Kawalekar OU, Ghassemi S, Milone MC. CAR T cell immunotherapy for human cancer. Science. 2018;359:1361–5. 29567707 10.1126/science.aar6711

[77] Ingram DA, et al. Lymphoproliferative defects in mice lacking the expression of neurofibromin: functional and biochemical consequences ofNf1 deficiency in T-cell development and function. Blood. 2002;100:3656–62. 12393709 10.1182/blood-2002-03-0734

[78] Larson K, et al. Pembrolizumab achieves a complete response in an NF-1 mutated, PD-L1 positive malignant peripheral nerve sheath tumor: a case report and review of the benchmarks. J Immunother Hagerstown Md. 2022;1997(45):222–6. 10.1097/CJI.0000000000000410 35020691

[79] Murray PJ. Macrophage Polarization. Annu Rev Physiol. 2017;79:541–66. 27813830 10.1146/annurev-physiol-022516-034339

[80] Boal LH, et al. Pediatric PK/PD phase I trial of pexidartinib in relapsed and refractory leukemias and solid tumors including neurofibromatosis type I related plexiform neurofibromas. Clin Cancer Res Off J Am Assoc Cancer Res. 2020;26:6112–21. 10.1158/1078-0432.CCR-20-1696 PMC7909006 32943455

[81] Fernández-Rodríguez J, et al. A high-throughput screening platform identifies novel combination treatments for Malignant Peripheral Nerve Sheath Tumors. Mol Cancer Ther. 2022;21:1246–58. 35511749 10.1158/1535-7163.MCT-21-0947 PMC9256801

[82] Deyle DR, Escobar DZ, Peng K-W, Babovic-Vuksanovic D. Oncolytic measles virus as a novel therapy for malignant peripheral nerve sheath tumors. Gene. 2015;565:140–5. 25843626 10.1016/j.gene.2015.04.001

