# Correction to: Past, Present, and Future Therapeutic Strategies for NF‑1‑Associated Tumors

**DOI:** 10.1007/s11912-024-01581-y

**Published:** 2024-07-19

**Authors:** Brian Na, Shilp R. Shah, Harish N. Vasudevan

**Affiliations:** 1 Department of Neurology, UCLA Neuro-Oncology Program, University of California Los Angeles, Los Angeles, CA 90095 USA; 2 Samueli School of Engineering, University of California Los Angeles, Los Angeles, CA 90095 USA; 3 Department of Radiation Oncology, University of California San Francisco, San Francisco, CA 94143 USA; 4 Department of Neurological Surgery, University of California San Francisco, San Francisco, CA 94143 USA

**Correction to: Current Oncology Reports (2024) 26:706–713**

10.1007/s11912-024-01527-4

The original version of this article unfortunately contained a mistake in the wording surrounding the ‘RAF inhibitor’ section. The 2nd sentence should be modified with the following.

**RAF Inhibition**

RAF inhibition, the most proximal downstream signaling protein from RAS, has been studied extensively in *NF1*-mutant tumors. First-generation RAF inhibitors selectively targeting the BRAFV600E mutation show minimal efficacy, with resistance occurring within 6 to 7 months [26, 27]. Accordingly, pan-RAF inhibitors have been developed to address these challenges. Tovorafenib in particular has been investigated in a Phase 2 trial FIREFLY-1 (NCT04775485) for recurrent BRAF-altered pediatric LGGs and demonstrated a meaningful radiographic response, although it is important to note this was not in *NF1* mutant tumors, and further work is necessary to determine whether RAF inhibition alone or in combination with other agents may be useful in the NF-1 setting (NCT04775485) [28•].

The original article has been corrected.

